# Supplementary material for: Multi-photon near-infrared emission saturation nanoscopy using upconversion nanoparticles
Source: Nat Commun. 2018 Aug 17;9:3290. doi: 10.1038/s41467-018-05842-w (PMC6098146; doi:10.1038/s41467-018-05842-w)
Supplement: Supplementary file 1 — Supplementary Information [file 41467_2018_5842_MOESM1_ESM.pdf]

Supplementary Information

Multi-photon near-infrared emission saturation nanoscopy using upconversion nanoparticles

Chaohao Chen[1†], Fan Wang[1†*], Shihui Wen[1], Qian Peter Su[1], Mike C.L. Wu[2], Yongtao Liu[1], Baoming Wang[1], Du Li[1], Xuchen Shan[1], Mehran Kianinia[1], Igor Aharonovich[1], Milos Toth[1], Shaun P. Jackson[2], Peng Xi[3], Dayong Jin[1,4*]

[1]Institute for Biomedical Materials and Devices (IBMD), Faculty of Science, University of Technology Sydney, NSW 2007, Australia.

[2]Heart Research Institute, and Charles Perkins Centre, University of Sydney, Camperdown NSW 2006, Australia.

[3]Department of Biomedical Engineering, College of Engineering, Peking University, Beijing 100871, China.

[4]ARC Research Hub for Integrated Device for End-user Analysis at Low-levels (IDEAL), Faculty of Science, University of Technology Sydney, NSW, 2007, Australia.

[†]These authors contributed equally.

[*]Correspondence to fan.wang@uts.edu.au, dayong.jin@uts.edu.au

Supplementary Note 1 Energy densities for the probes

The most common probes to achieve STED[1,2,3] and GSD[4] (Supplementary Table 1) includes fluorescent proteins, quantum dots, semiconductor nanowires and UCNPs. Supplementary Table 1 summaries the key parameters for these probes. To compare the maximum laser induced energy dosage, required by different probes to achieve nanoscopy through deep tissue, we calculated the energy density (I_Q) of both excitation and depletion laser during excitation time of 200 fs through 100 μm skin tissue.

We can calculate the required I_Q according to:

$$R_{\mathrm{tr}} = e^{-\alpha_\lambda l} \tag{1}$$

$$I_Q = \frac{P r_{\mathrm{o}} t_\tau}{A f t_{\mathrm{p}} R_{\mathrm{tr}}} \tag{2}$$

Where R_{tr} is the transmition ratio of an electromagnetic wave penetrating a material (Beer's law); P is the beam power; r_{o} is the loss rate of the objective lens; t_τ is the exposure time; A is the area of the focused laser spot; f is the pulse frequency; t_{p} is the pulse duration; α_λ is the attenuation coefficient; λ is the wavelength; l is the path length. Note that the value of f and t_{p} is 1 for CW laser. The loss rate of the laser through the objective lens is based on our current system (r_{o} = 0.43), and the area of the focused laser spots are $A_{\mathrm{gau}} = 3.76 \times 10^{-9}$ cm^{-2} for Gaussian beam and $A_{\mathrm{dou}} = 7.81 \times 10^{-9}$ cm^{-2} for Doughnut beam. The exposure time is 200 fs, and the path length is 100 μm.

Supplementary Table 1 | Key parameters of various imaging modalities for deep tissue. The summarized key parameters to calculate the energy densities for deep tissue imaging, including excitation wavelength, emission wavelength, frequency, pulse duration, power intensity etc. 1PE, one-photon excitation; 2PE, two-photon excitation; MP, Multi-photon excitation; QD, quantum dots; FP, fluorescence protein; SEMI, semiconductor nanowires segments.

**Nanoscope**	**Probe**	**λ_{ex} (nm)**	**λ_{dep} (nm)**	***f* (MHz)**	**Pulse duration t_p (ps)**	**Power intensity *I* (mW)**	**Energy intensity I_Q (J cm^{-2})**
STED[1]	1PE-QD	628	-	38	1.2	0.05	7.9×10^{-5}
		-	775	38	1200	150	
STED[2]	2PE-FP	850	-	76	0.13	2.7	9.4×10^{-3}
		-	580	76	200	4.4	
STED[3]	MP-UCNP	980	-	CW	-	1	7.6×10^{-7}
		-	808	CW	-	40	
GSD[4]	1PE-SEMI	700	Non	80	5	5	2.5×10^{-4}
NIRES	MP-UCNP	980	Non	CW	-	75	1.2×10^{-6}

Supplementary Note 2 Resolution of NIRES nanoscopy

Simulation Method. The approximate function of optical resolution in a STED or GSD microscope has been derived, releasing the famous square root law[5]. The full-width at half maximum (FWHM) of STED point spread function (PSF) with fluorophore that contain two energy level (Supplementary Fig. 1a) can be represented as:

$$\Delta \mathrm{x} = \frac{h_0}{\sqrt{\varsigma}} \tag{3}$$

Here h_0 denotes the FWHM of confocal PSF. $\varsigma = I_{\mathrm{STED}}^{\mathrm{Max}}/I_S$ denotes the saturation factor. I_{S} is referred to the saturation intensity where the emission intensity decreases to half of maximum. I_{STED}^{Max} represent the maximum amplitude of STED beam profile.

The validity of this function of FWHM in STED actually can be extended for two-photon STED and two-photon negative GSD where the fluorophore is excited by absorbing two photons with energy below its band gap, through modifying the function of saturation intensity as $I_{\mathrm{S}} = \sqrt{k_{\mathrm{BA}}/\sigma_{\mathrm{TPA}}}$. Here k_{BA} is the carrier transition rate from excited state B to ground state A, σ_{TPA} denotes the molecular cross section with two-photon absorption.

The NIRES nanoscopy in this paper has similar physical process with two-photon negative GSD but may not be able use the existing function of resolution (Supplementary Equation 3). UCNP has much more complex rate transition system (Supplementary Fig. 1b) than the aforementioned two-level system, with rate equation shown as:

$$\frac{dn_1}{dt} = -c_1 n_1 n_{S2} + a_{21} w_2 n_2 + a_{31} w_3 n_3 + a_{41} w_4 n_4 + a_{51} w_5 n_5 - k_{41} n_1 n_4 - k_{31} n_3 n_1 - k_{51} n_5 n_1$$

$$\frac{dn_2}{dt} = c_1 n_1 n_{S2} - c_2 n_2 n_{S2} - a_{21} w_2 n_2 + a_{32} w_3 n_3 + a_{42} w_4 n_4 + a_{52} w_5 n_5 + k_{41} n_1 n_4 + 2k_{31} n_1 n_3$$

$$\frac{dn_3}{dt} = c_2 n_2 n_{S2} - c_3 n_3 n_{S2} - (a_{31} + a_{32}) w_3 n_3 + a_{43} w_4 n_4 + a_{53} w_5 n_5 + 2k_{51} n_5 n_1 + k_{41} n_4 n_1 - k_{31} n_3 n_1$$

$$\frac{dn_4}{dt} = c_3 n_3 n_{S2} - c_4 n_4 n_{S2} - (a_{43} + a_{42} + a_{41}) w_4 n_4 + a_{54} w_5 n_5 - k_{41} n_1 n_4$$

$$\frac{dn_5}{dt} = c_4 n_4 n_{S2} - (a_{54} + a_{53} + a_{52} + a_{51}) w_5 n_5 - k_{51} n_1 n_5$$

$$\frac{dn_{S2}}{dt} = P_{980} n_{S1} - w_{S2} n_{S2} - (c_1 n_1 + c_2 n_2 + c_3 n_3 + c_4 n_4) n_{S2}$$

Here we simplify the energy level which involves two energy levels associated with the sensitizer Yb^{3+} ions and five associated levels with the activator Tm^{3+} ions; n_{S1}, n_{S2}, n_1, n_2, n_3, n_4 and n_5 are the populations of ions on energy levels of $^2F_{7/2}$, $^2F_{5/2}$, $^3H_6$, $^3H_5/^3F_4$ and $^3F_{2,3}/^3H_4$ respectively; c_i is the energy transfer ratio between Yb^{3+} on the excited level and Tm^{3+} both on the ground and the intermediate levels; k_{ij} is the cross-relaxation coefficients between the state *i* and *j*; a_{ij} is the branching ratio from energy level *i* to *j*; W_i is the intrinsic decay

rate of Tm^{3+} on level *i*; *P* is the absorption rate of Yb^{3+}; n_3 ($^3H_4$) is the excited state used in this paper; the fitting parameters are summarized in Supplementary Table 2. Therefore, the resultant carrier number (emission intensity) function of excitation power is significant different with that for two-photon excited two-level system as shown in Supplementary Fig. 1c, which further results in a different function of resolution for NIRES nanoscopy.

Supplementary Table 2 | The values of key constants and rate parameters used in the simulations[3]. c_i is the energy transfer ratio between Yb^{3+} on the excited level and Tm^{3+} both on the ground and the intermediate levels; k_{ij} is the cross-relaxation coefficients between the state *i* and *j*; a_{ij} is the branching ratio from energy level *i* to *j*; w_i is the intrinsic decay rate of Tm^{3+} on level *i*; P_{980} is the absorption rate of Yb^{3+}.

w_2 (s^{-1})	w_3 (s^{-1})	w_4 (s^{-1})	w_5 (s^{-1})	w_{s2} (s^{-1})
63000	20000	15000	33000	8000
a_{51}	a_{52}	a_{53}	a_{54}	
0.24	0.23	0.2	0.33	
a_{41}	a_{42}	a_{43}	a_{31}	a_{32}
0.18	0.24	0.58	0.27	0.73
c_1 (s^{-1})	c_2 (s^{-1})	c_3 (s^{-1})	c_4 (s^{-1})	
62000	50000	70000	5000	
k_{31} (s^{-1})	k_{41} (s^{-1})	k_{51} (s^{-1})	P_{980} (s^{-1})	
140000	185000	500000	280000	

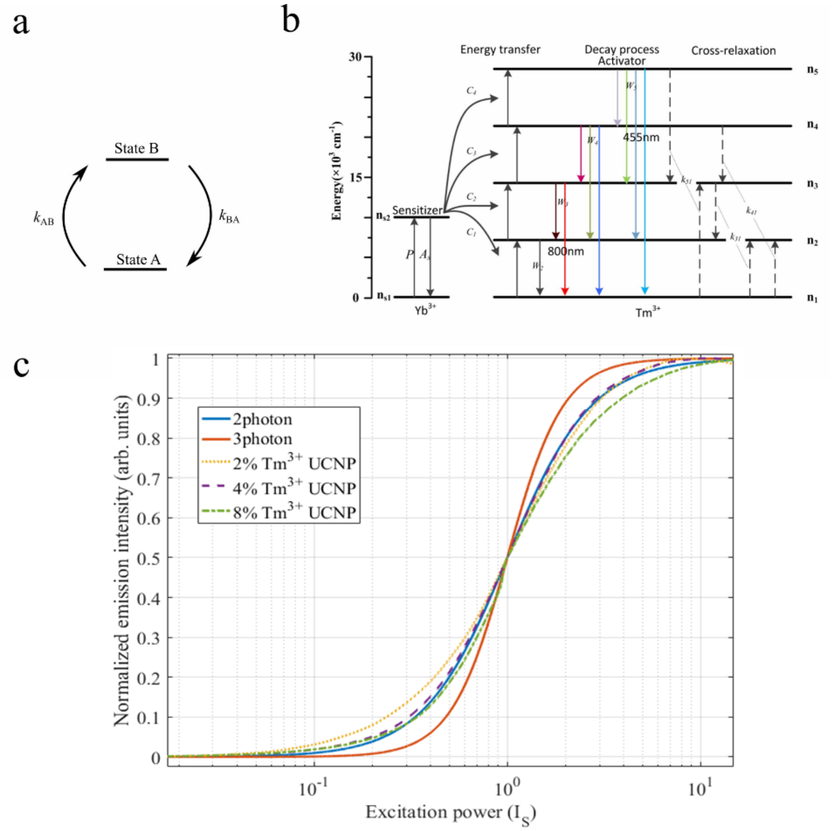

Supplementary Figure 1 | Rate transition system of UNCPs. (**a**) The energy level diagram of two levels system, with excited state B and ground state A. (**b**) The energy level diagram of Tm^{3+} and Yb^{3+} doped UCNP. (**c**) Simulated excitation power dependent emission intensity for two-photon excited two energy level system (labeled as 2 photon), three-photon excited two energy level system

(labeled as 3 photon) and UCNPs with 2%, 4% and 8% Tm^{3+} doping. It is noted that the excitation power is normalized to saturation intensity (I_S) where the emission intensity is dropped by half.

According to the description in maintext and shown in Supplementary Fig. 1c and Fig. 6c, 4% Tm^{3+} doped UCNPs have larger onset value than 2% and 3% Tm^{3+} doped UCNPs, and smaller I_{MAX} value than 6% and 8% Tm^{3+} doped UCNPs. Therefore 4% is the optimized doping concentration for NIRES. Note that 4% Tm^{3+} doped UCNPs have a similar power dependent curve with that for two-photon excited two-level system, which indicates that the best resolution they can achieve is same. It is also notable that even though the best resolution for 4% Tm^{3+} will be similar with that for two-photon excited two-level system, 4% Tm^{3+} still can produce much better resolution with limited excitation power as it has much smaller I_S.

Following a similar derivation as in previous works[5,6], we define the effective PSF of the NIRES as:

$$\begin{cases} h_{\mathrm{eff}}(x) = h_{\mathrm{em}}(x) \times h_{\mathrm{c}}(x) \\ h_{\mathrm{em}}(x) = \eta(i) \times h_{\mathrm{exc}}(x) \end{cases}$$

Here $h_{em}(x)$ is the PSF of emission; $h_{exc}(x)$ is the PSF of excitation beam (donut beam for NIRES); η is excitation power dependent emission intensity curve; $h_c(x)$ is the PSF of the confocal collection system. The FWHM of the intensity dip in h_{eff} represents the resolution for NIRES nanoscopy.

Experimental Result and influence of particle size. The experimentally measured PSF (h_{exp}) of NIRES is the convolution between the h_{eff} and the spatial profile (h_{UCNP}) of nanoparticle as below:

$$h_{\mathrm{exp}} = h_{\mathrm{eff}} * h_{\mathrm{UCNP}}$$

The deconvolution process on h_{exp} results in a measured h_{eff} in which the FWHM of the dip represent the resolution of NIRES. In this paper the resolution is calculated through deconvolution of experimental measured PSF.

The theoretical simulation of h_{exp} for different particle size is shown in Supplementary Fig. 2a, where the PSF of UCNP with size 0nm indicates the h_{eff}. The result indicates that larger particle size leads to larger value of the FWHM of h_{exp} (Supplementary Fig. 2b) and the dip height of h_{exp} (Supplementary Fig. 2c), which in turn offers lower resolution. It is notable that, the FWHM of the dip of h_{exp} can be smaller than the particle' size (Supplementary Fig. 2b) when the FWHM of h_{eff} is smaller than the particle's size, which stems from the donut shape PSF of h_{eff}. If the shape of h_{eff} is a Gaussian function, the FWHM of the PSF after convolution is always larger than the size of particle.

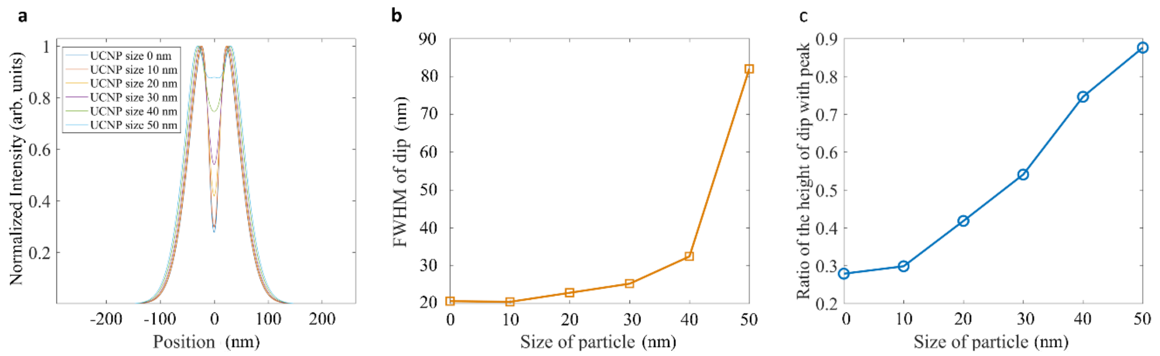

Supplementary Figure 2 | Theoretical simulation of image of single UCNP by NIRES. (**a**) The PSF of UCNP with size varying from 0nm to 50nm. (**b**) The FWHM of the dip in h_{exp} for UCNPs with different size. (**c**) The ratio of the height value of the dip with the peak value in h_{exp} for UCNPs with different size. The UCNPs with 4% Tm^{3+}, 20% Yb^{3+} are used in this simulation. The excitation peak intensity is 100 times larger than the saturation intensity of UCNP.

Supplementary Note 3 Experimental setup

All the measurements are performed on a home-built microscopy system equipped with a 3-axis closed-loop piezo stage (stage body MAX311D/M, piezo controller BPC303; Thorlabs) and a vortex phase plate (VPP, VPP-1a, RPC Photonics). Supplementary Fig. 3 shows the schematic drawing of the experimental setup, where UCNPs are excited by a polarization-maintaining single-mode fiber-coupled 980 nm diode laser (BL976-PAG900, controller CLD1015, Thorlabs). The first half wave plate (HWP, WPH05M-980, Thorlabs) and polarized beam splitter (PBS, CCM1-PBS252/M, Thorlabs) are employed to precisely adjust the excitation power by rotating HWP electronically. The purpose of the second HWP was to turn the polarization from horizontal (P polarized) to vertical (S polarized). A doughnut-shaped point spread function (PSF) at the focal plane is generated by a VPP. Confocal scanning is acquired without the VPP via the auxiliary two flexible mirrors as shown in the dotted portion of Supplementary Fig. 3. After collimation, the excitation beam is reflected by the short-pass dichroic mirror (DM, T875spxrxt-UF1, Chroma), and focused through a high numerical aperture objective (UPlanSApo, 100×/1.40 oil, Olympus) to the sample slide. A quarter-wave plate (QWP, WPQ05M-980, Thorlabs) is adopted to transform the excitation beam from linear polarization to circular polarization to obtain optical super-resolution images. Photoluminescence is collected by the same objective and split from the excitation beams by DM. The emission signals were filtered by band pass filters (BPF, ET805/20M, Chroma) or short pass filter (SPF, FF01-842/SP-25, Semrock), and coupled into multi-mode fiber (MMF, M42L02, Thorlabs), then detected by a single-photon counting avalanche photodiode (SPAD, SPCM-AQRH-14-FC, Excelitas). The MMF could also be switched to a miniature monochromator (iHR550, Horiba) for measuring upconversion emission spectra. Typical excitation powers for the recording of super-resolution NIRES images varied from 5 mW to 100 mW. All powers were measured at the back aperture of the objective lens. Pixel dwell times were adjusted to be ~3 ms.

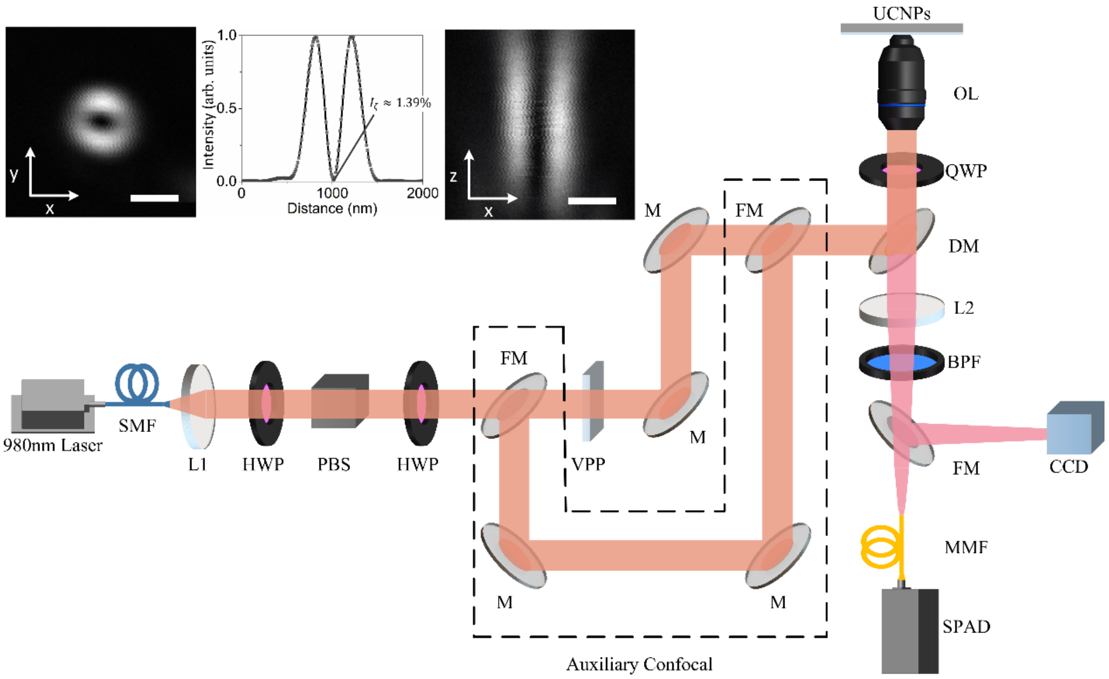

Supplementary Figure 3 | Experimental setup for NIRES nanoscopy (SMF, single-mode fiber; MMF, multi-mode fiber; L1, collimation lens; L2, collection lens; HWP, half-wave plate; QWP, quarter-wave plate; PBS, polarized beam splitter; VPP, vortex phase plate; M, mirror; FM, flexible mirror; DM, dichroic mirror; OL, objective lens; BPF, band pass filter; SPAD, single-photon avalanche diode; CCD, charge coupled device). The dotted portion is designed for auxiliary confocal with two flexible mirrors to bypass the VPP in the main optical path. Inset shows point spread function (PSF) of the NIRES is measured by scattering of a 100 nm gold bead in reflection (path not shown). The I_ζ (ratio value of the intensity at the doughnut center to the max intensity of the beam) is measured as 1.39%. Scale bars: 500nm.

Supplementary Note 4 Materials

$YCl_3 \cdot 6H_2O$ (99.99%), $YbCl_3 \cdot 6H_2O$ (99.99%), $TmCl_3 \cdot 6H_2O$ (99.99%), NH_4F (99.99%), NaOH (99.9%), oleic acid (OA, 90%), and 1-octadecene (ODE, 90%) were purchased from Sigma-Aldrich.

Synthesis of $NaYF_4$: Yb, Tm nanocrystals. $NaYF_4$:Yb,Tm nanocrystals with different Tm doping (from 2% to 8%) were synthesized according to our previously reported method[3,7]. Taken $NaYF_4$: 20% Yb, 2% Tm as an example, 1 mmol $RECl_3$ (RE = Y, Yb, Tm) with the molar ratio of 78:20:2 were added to a flask containing 6 ml OA and 15 ml ODE. The mixture was heated to 170 °C under argon for 30 min to obtain a clear solution and then cooled down to about 50 °C, followed by the addition of 5 mL methanol solution of NH_4F (4 mmol) and NaOH (2.5 mmol). After stirring for 30 min, the solution was heated to 80 °C under argon for 20 min to remove methanol, and then the solution was further heated to 310 °C for another 90 min. Finally, the reaction solution was cooled down to room temperature, and nanoparticles were precipitated by ethanol and washed with cyclohexane, ethanol and methanol for 3 times to get the $NaYF_4$: 20% Yb, 2% Tm nanoparticles.

To get the nanoparticles with desired size or core-shell structure, layer-by-layer epitaxial growth has been employed. The shell precursors preparation was similar with that for the core nanoparticles synthesis, until the

step where the reaction solution was slowly heated to 150 °C and kept for 20 min. Instead of further heating to 300 °C to trigger nanocrystal growth, the solution was cooled down to room temperature to yield the shell precursors. For epitaxial growth, 0.15 mmol as-prepared core nanocrystals were added to a containing 6 ml OA and 6 ml ODE. The mixture was heated to 170 °C under argon for 30 min, and then further heated to 300 °C. Next, 0.25 ml as prepared shell precursors were injected into the reaction mixture and ripened at 300 °C for 4 min, followed by the same injection and ripening cycles for several times to get the nanocrystals with the desired size. Finally, the slurry was cooled down to room temperature and the formed nanocrystals were purified according to the above procedure.

Characterization Techniques. The morphology of the formed materials was characterized *via* transmission electron microscopy (TEM) imaging (Philips CM10 TEM with Olympus Sis Megaview G2 Digital Camera) with an operating voltage of 100 kV. The samples were prepared by placing a drop of a dilute suspension of nanocrystals onto copper grids.

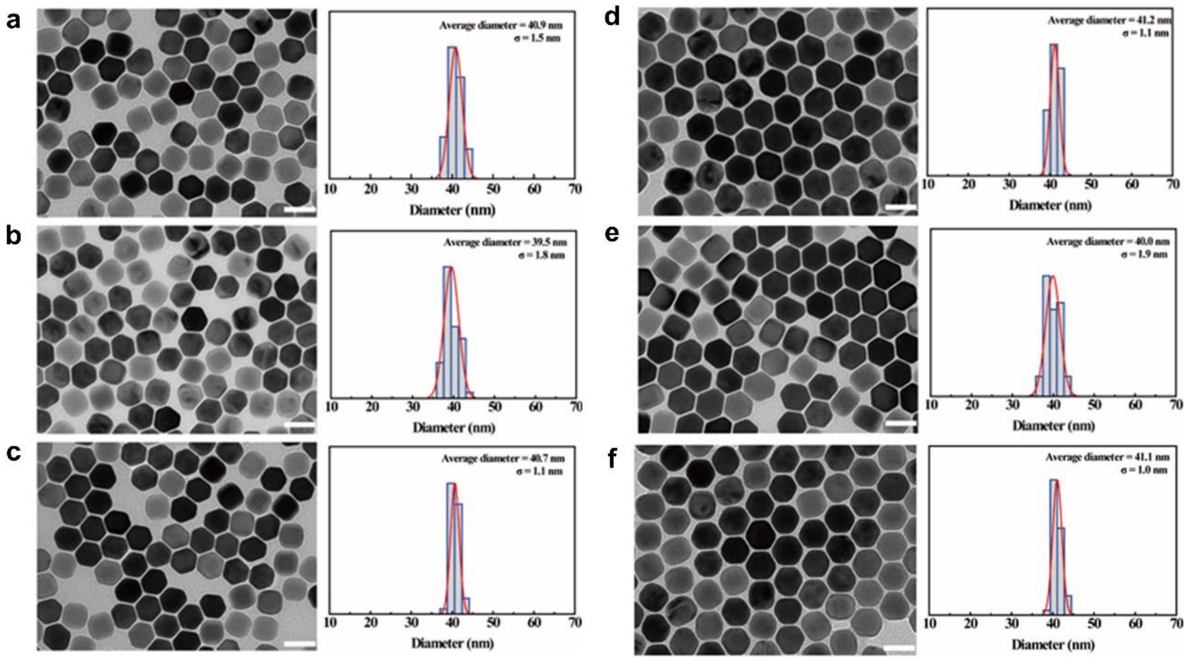

Supplementary Figure 4 | TEM images (left) and size distribution histograms (right) of the nanoparticles. (**a**) $NaYF_4$: 20% Yb, 2% Tm. (**b**) $NaYF_4$: 20% Yb, 3% Tm. (**c**) $NaYF_4$: 20% Yb, 4% Tm. (**d**) $NaYF_4$: 20% Yb, 6% Tm. (**e**) $NaYF_4$: 20% Yb, 8% Tm. (**f**) $NaYF_4$: 40% Yb, 4% Tm. Scale bar is 50 nm.

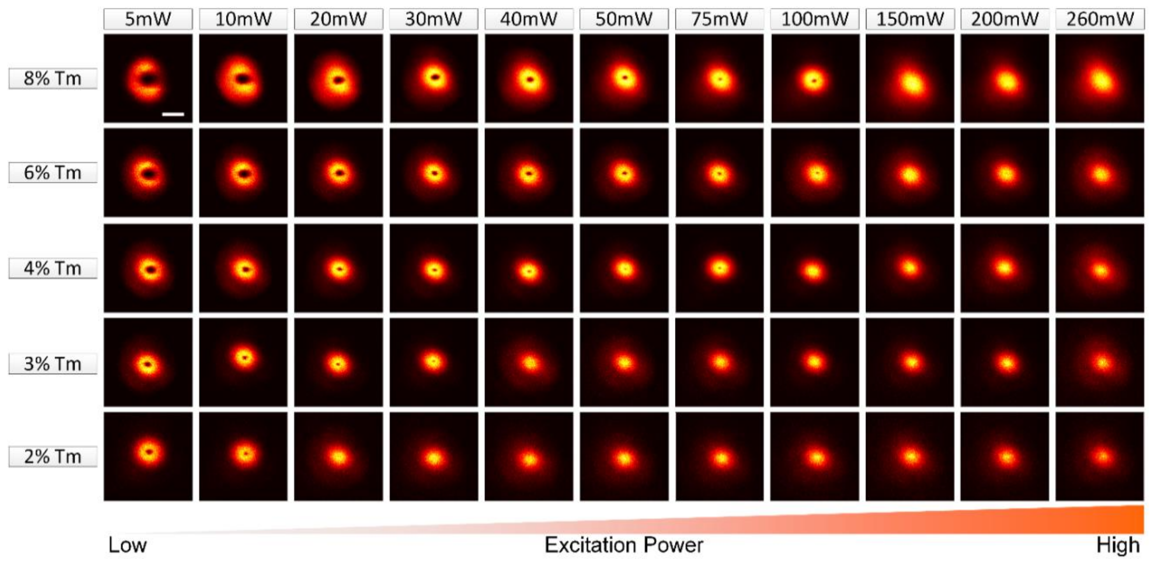

Supplementary Figure 5 | The acquired images of UCNP under different situation. $NaYF_4$: 20% Yb^{3+}, x% Tm^{3+}, ~40 nm in diameter, x= 2, 3, 4, 6, 8. All the laser powers are measured at the back aperture. Pixel dwell time, 3 ms; pixel size, 10 nm. Scale bar is 500 nm.

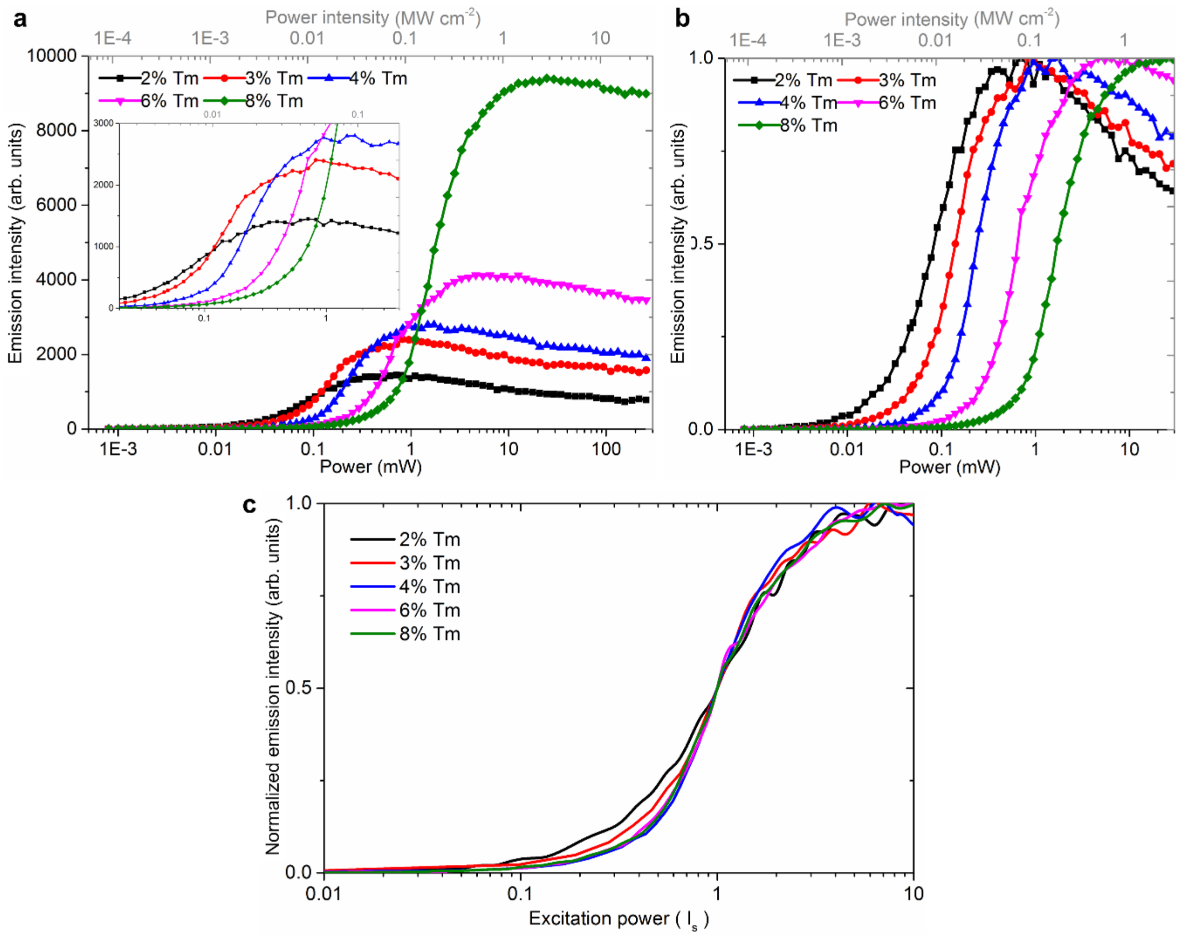

Supplementary Figure 6 | The saturation intensity curve of the 800 nm emissions from UCNPs. (**a**) 800 nm emission saturation curves obtained for a single UCNP ($NaYF_4$: 20% Yb^{3+}, x% Tm^{3+} nanoparticles, x=2, 3, 4, 6 and 8) under 980 nm excitation. (**b**) Normalized emission saturation curve to the maximum intensity. (**c**) The emission saturation curve is normalized to saturation intensity (I_S) where the emission intensity is dropped by half.

Supplementary Note 5 Preparation of HeLa cell samples and internalization of UCNPs

The human cervical cancer (HeLa) cells were obtained from the American Type Culture Collection (ATCC®CCL-2™). The cells were incubated in Dulbecco's High Glucose Modified Eagle's Medium (DMEM) contained with 10% fetal bovine serum (FBS), and incubated at 37 °C and 5% CO_2.

For the internalization of UCNPs, $5*10^5$ HeLa cells were seeded on a glass coverslip in a 6 wells plate with 2% FBS for 24 h. Then, 30 μg/ml UCNPs coated with home-made polymer were put into the well to allow the internalization for 2 h, followed by phosphate buffer saline washing for 3 times, 5 minutes per time, to make sure removal of all UCNPs that were not internalized. Then, the cover slide was mounted on a glass slide. In order to avoid the moving of nanoparticle in cell, the sample was put in 4 °C for 10 minutes before the microscopy measurement.

We verify the power of NIRES modality by resolving single 40 nm UCNPs inside HeLa cells Supplementary Fig. 7a shows the wide field imaging of multiple UCNPs inside HeLa cell. Supplementary Fig. b & c display the large confocal scanning at center region with size (10 μm × 10 μm) and (6 μm × 6 μm), respectively. Supplementary Fig. 7d shows that NIRES can clearly distinguish UCNPs within a diffraction limit area, which cannot be resolved by the conventional confocal microscope (Supplementary Fig. 7c). Same as SAC method[8], higher excitation power will raise up the dip in the PSF of emission for NIRES to the maximum point of PSF according to the saturation curve (Fig. 2b), thereby switching NIRES into a confocal microscopy obtaining "confocal image". The subtraction of the "confocal image" with respective to negative NIRES image will further provide a positive NIRES image.[1] This results in a positive NIRES image, shown in Supplementary Fig. 7g. The intensity line profiles (Supplementary Fig. 7h) show the distance between the two UCNPs is 280 nm.

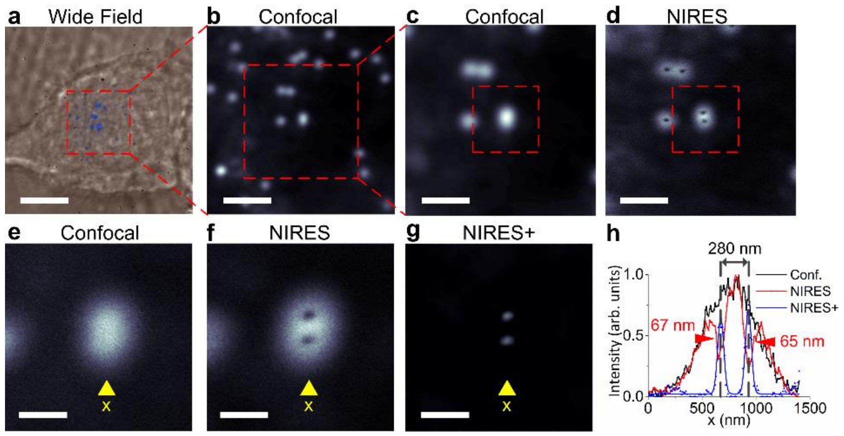

Supplementary Figure 7 | NIRES super resolution imaging of single UCNPs inside the HeLa cell. (**a**) Bright field and fluorescence wide field image of UCNPs up taken by a HeLa cell. (**b**) and (**c**) Confocal images of areas of (10 μm × 10 μm) and (6 μm × 6 μm) respectively. (**d**) NIRES image showing two UCNPs in a diffraction limit area. Zoom-in confocal image (**e**) and NIRES image (**f**). (**g**) The positive NIRES image by subtracting (**f**) from (**e**). (**h**) Cross-section line profiles of UCNPs in confocal image (**e**), raw NIRES image (**f**), and positive NIRES image (**g**). Pixel dwell time for confocal and NIRES is 3 ms. The Pixel size for confocal and NIRES is 10 nm. The scale bar is 7.5μm in (**a**), 2.5 μm in (**b**), 1.5 μm in (**c**) and 500 nm in (**d**) – (**g**).

Supplementary Note 6 Tissue sample preparation

Mice post euthanasia with an injection of lethal dose of Xylazine and Ketamine mixture, the mice were transcardially perfused with saline to remove blood content. Brain, kidney and liver tissue samples were collected and fixed in 4% PFA overnight at 4 °C, and sectioned into 100 µm, 150 µm, 200 µm in thickness, using an automated vibratome (Leica VT1200 S). The brain, kidney and liver sections were then mounted in glycerol containing 0.05 mg/ml UCNPs for NIRES imaging. All procedures performed on mice were approved by Animal Care and Use Committee, the University of Sydney Animal Ethics Committee (2017/1197).

To demonstrate the advance of the longer wavelength for deep tissue penetration (Supplementary Fig. 8), we use the UV-Vis spectrophotometer (Cary 60 UV-Vis, Agilent Technologies) to measure the light absorption through 50 µm and 100 µm liver, brain, and kidney tissue slice samples, respectively (Supplementary Fig. 9). As can be seen, the extinction rates of these tissues decrease in general with increasing the light wavelength in visible and near-IR region.

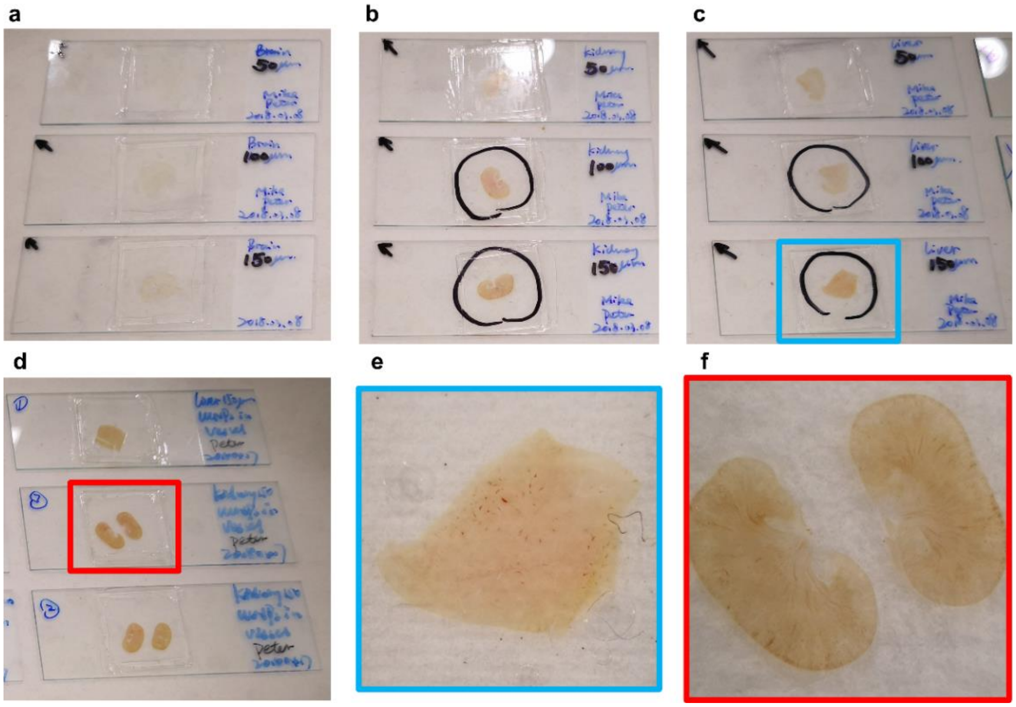

Supplementary Figure 8 | Photographs of different mouse tissue slices on glass slides. (**a**) Brain tissue slices. (**b**) Kidney tissue slices. (**c**) Liver tissue slices. (**d**) Kidney and liver tissue with blood slices. (**e**) Zoom-in the tissue slice of blue square in (**c**). (**f**) Zoom-in the tissue slice of red square in (**d**).

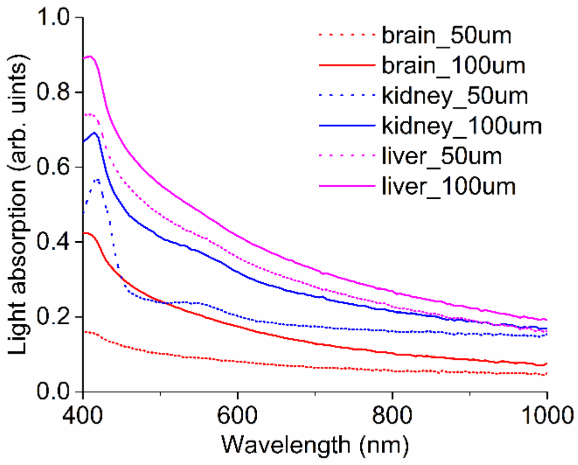

Supplementary Figure 9 | UV–vis absorption spectra of tissue slice. 50 µm and 100 µm thickness of live, brain, and kidney tissue slice samples are used for the measurement of tissue light absorption.

Supplementary Table 3 | FWHM of 455 nm, 800 nm emission confocal and NIRES at different depth of a liver tissue slice. 455 nm, FWHM of confocal images from 455 nm emission; 800nm, FWHM of confocal images from 800 nm emission; NIRES, FWHM of the corresponding NIRES images.

	**0 µm**	**15 µm**	**55 µm**	**75 µm**	**93 µm**
**455 nm**	321.2 ± 15.7 nm	329.8 ± 24.3 nm	341.5 ± 18.9 nm	359.4 ± 14.5 nm	400.7 ± 16.4 nm
**800 nm**	445.0 ± 10.5 nm	423.3 ± 27.5 nm	481.7 ± 34.3 nm	475.0 ± 33.5 nm	488.3 ± 17.6 nm
**NIRES**	49.6 ± 11.1 nm	42.4 ± 6.2 nm	42.4 ± 7.2 nm	48.0 ± 7.3 nm	38.2 ± 14.3 nm

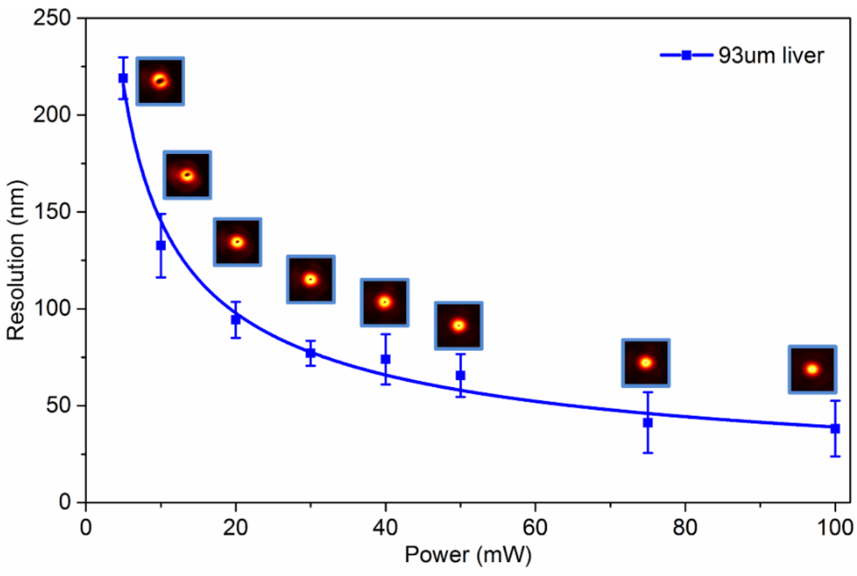

Supplementary Figure 10 | FWHM as a function of the excitation power inside liver tissue. The UCNP is fixed at 93 µm depth inside liver tissue. All the laser powers are measured at the back aperture. Error bars are defined as s.d.. Pixel dwell time, 3 ms; pixel size, 10 nm.

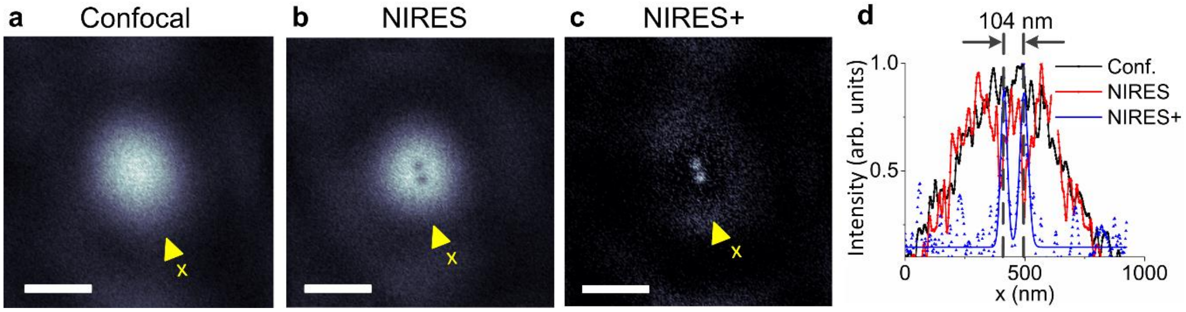

Supplementary Figure 11 | Resolved two particles with distance below the diffraction limit in 65 μm depth inside liver tissue. (**a**) Confocal scanned image (2 μm × 2 μm) of the UCNPs sample. (**b**) The same position obtained by NIRES, with distinct UCNPs that could not be separated by confocal. (**c**) The positive NIRES sub-diffraction image by (**b**) subtracted from (**a**). (**d**) Cross-section line profile of UCNPs in raw NIRES image (**b**), subtracted image (**c**) and confocal image (**a**). Pixel dwell time, 3 ms; pixel size, 10 nm. Scale bar, 500 nm.

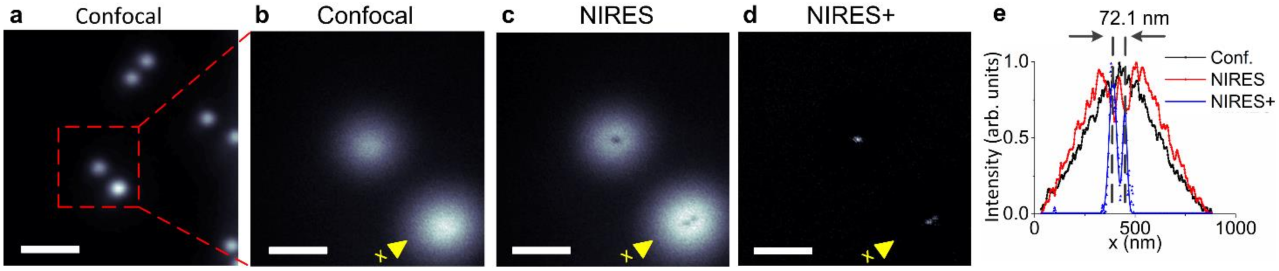

Supplementary Figure 12 | Resolved two particles with distance below the diffraction limit. (**a**) Confocal scanned image (6 μm×6 μm) of the UCNPs sample. (**b**) Confocal image enlarge the red dotted square in (**a**). (**c**) The same position obtained by NIRES, with distinct UCNPs that could not be separated by confocal. (**d**) The positive NIRES sub-diffraction image by (**c**) subtracted from (**b**). (**e**) Cross-section line profile of UCNPs in raw NIRES image (**c**), subtracted image (**d**) and confocal image (**b**). Pixel dwell time, 3 ms; pixel size, 10 nm. Scale bar, 1.5 μm in (**a**); 500 nm in (**b**) – (**d**).

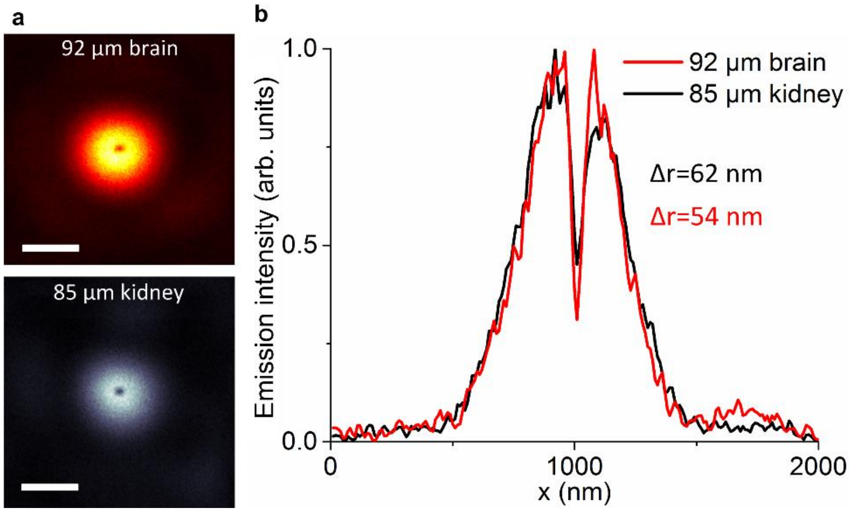

Supplementary Figure 13 | NIRES images in deep mouse brain and kidney tissue. (**a**) NIRES image at 92 μm depth inside of brain tissue (upper) and 85 μm depth inside kidney tissue (bottom), respectively. (**b**) The corresponding cross section profile lines. Pixel dwell time, 3 ms; pixel size, 10 nm. Scale bar, 500 nm.

Supplementary Reference

1. Hanne, J. *et al.* STED nanoscopy with fluorescent quantum dots. *Nat. Commun.* **6,** 7127 (2015).

2. Li, Q., Wu, S. S. H. & Chou, K. C. Subdiffraction-limit two-photon fluorescence microscopy for GFP-tagged cell imaging. *Biophys. J.* **97,** 3224–3228 (2009).

3. Liu, Y. *et al.* Amplified stimulated emission in upconversion nanoparticles for super-resolution nanoscopy. *Nature* **543,** 229–233 (2017).

4. Oracz, J. *et al.* Ground State Depletion Nanoscopy Resolves Semiconductor Nanowire Barcode Segments at Room Temperature. *Nano Lett.* **17,** 2652–2659 (2017).

5. Hell, S. W. Toward fluorescence nanoscopy. *Nat. Biotechnol.* **21,** 1347–1355 (2003).

6. Zhao, G., Kuang, C., Ding, Z. & Liu, X. Resolution enhancement of saturated fluorescence emission difference microscopy. *Opt. Express* **24,** 23596–23609 (2016).

7. Liu, D. *et al.* Three-dimensional controlled growth of monodisperse sub-50 nm heterogeneous nanocrystals. *Nat. Commun.* **7,** 10254 (2016).

8. Zhao, G. *et al.* Saturated absorption competition microscopy. *Optica* **4,** 633 (2017).
